# Differential Associations of Oxidative Biomarkers with Symptomatic and Systolic Severity in Heart Failure

**DOI:** 10.3390/medicina62061108

**Published:** 2026-06-06

**Authors:** Aleksandra Arsić, Bojana Kisić, Vladan Perić, Ivana Stevanović, Ana Savić Radojević, Zoran Bukumirić, Ilija Dragojević, Marija Vasić, Martin Popević, Dragiša Rašić, Snežana Hadžistević

**Affiliations:** 1 Department of Medical Biochemistry, Military Hospital of the Ministry of Defense, 21000 Novi Sad, Serbia; 2 Institute of Biochemistry, Faculty of Medicine, University of Priština in Kosovska Mitrovica, 38220 Kosovska Mitrovica, Serbia; 3 Department of Internal Medicine, Faculty of Medicine, University of Priština in Kosovska Mitrovica, 38220 Kosovska Mitrovica, Serbia; 4 Medical Faculty of Military Medical Academy, University of Defense, 11000 Belgrade, Serbia; 5 Institute for Medical and Clinical Biochemistry, Faculty of Medicine, University of Belgrade, 11000 Belgrade, Serbia; 6 Department of Medical Statistics and Informatics, Faculty of Medicine, University of Belgrade, 11000 Belgrade, Serbia; 7 Institute of Medical Biochemistry, Military Medical Academy, 11000 Belgrade, Serbia; 8 Institute of Occupational Health, Military Medical Academy, 11000 Belgrade, Serbia; 9 Institute of Pharmacology, Faculty of Medicine, University of Priština in Kosovska Mitrovica, 38220 Kosovska Mitrovica, Serbia; s.hadzistevic@med.pr.ac.rs

**Keywords:** heart failure, oxidative stress, malondialdehyde, nitric oxide, natriuretic peptide

## Abstract

*Background and Objectives*: Oxidative stress is recognized as an important contributor to heart failure (HF) pathophysiology, but the relationships of individual oxidative and antioxidant biomarkers with symptomatic severity and systolic dysfunction remain insufficiently defined. This study examined circulating oxidative and nitrosative stress markers across New York Heart Association (NYHA) classes and left ventricular ejection fraction (LVEF) categories in HF and their associations with HF severity. *Materials and Methods*: In this case–control study, 85 patients with HF and 33 healthy controls were included. Malondialdehyde (MDA), nitrates and nitrites (NOx), superoxide dismutase (SOD), catalase (CAT), glutathione (GSH), sulfhydryl (SH) groups, and NT-proBNP were measured. Group differences were analyzed using the Kruskal–Wallis test with post hoc comparisons. Adjusted ordinal logistic regression models examined associations with NYHA class and LVEF category, and receiver operating characteristic (ROC) analysis evaluated discriminatory performance. *Results*: Compared with controls, all biomarkers differed significantly across NYHA classes and LVEF categories (all *p* < 0.001). In separate adjusted models, higher NOx, MDA, and NT-proBNP were associated with worse NYHA class and more impaired LVEF, whereas higher antioxidant marker levels were associated with lower odds of severe HF. In combined models, NOx remained independently associated with worse NYHA class (OR 1.07, 95% CI 1.04–1.11; *p* < 0.001), while MDA remained independently associated with more impaired LVEF (OR 1.02, 95% CI 1.00–1.03; *p* = 0.022). NT-proBNP showed the best discrimination for NYHA III/IV versus I/II (AUC 0.966), while among oxidative biomarkers NOx performed best for symptomatic severity (AUC 0.782) and MDA for LVEF ≤ 40% (AUC 0.751). *Conclusions*: HF is characterized by increased oxidative and nitrosative stress together with reduced antioxidant defense. NOx appears more closely related to symptomatic severity, whereas MDA appears more closely related to systolic dysfunction. However, NT-proBNP remained the strongest overall discriminator. NOx and MDA may provide complementary mechanistic information on redox imbalance across HF severity categories.

## 1. Introduction

Heart failure (HF) is a major public health challenge worldwide, affecting more than 64 million people and contributing substantially to morbidity, reduced functional capacity, poorer quality of life, and rising healthcare costs [1]. In the adult population, HF affects around 1–2% of individuals, with prevalence increasing markedly with age and exceeding 10% in those older than 70 years [1,2]. The European Society of Cardiology defines HF as a clinical syndrome caused by structural and/or functional cardiac abnormalities that impair ventricular filling or ejection, leading to elevated intracardiac pressures and/or inadequate cardiac output at rest or during exercise [2]. Its most common causes include ischemic heart disease, hypertension, valvular disorders, arrhythmias, and other myocardial or systemic conditions [2,3].

HF develops as a consequence of acute or chronic cardiac injury that reduces pump performance and triggers compensatory neurohormonal mechanisms, particularly activation of the renin–angiotensin–aldosterone system and the sympathetic nervous system. Although these responses may initially help maintain circulation, persistent activation contributes to adverse remodeling and progressive worsening of cardiac function [2,4]. In clinical practice, disease severity is commonly assessed using both symptom-based and objective parameters. The New York Heart Association (NYHA) classification remains the most widely used measure of symptom burden and functional limitation [5], while left ventricular ejection fraction (LVEF) is used to classify HF into preserved (HFpEF; LVEF ≥ 50%), mildly reduced (HFmrEF; LVEF 41–49%), and reduced ejection fraction (HFrEF; LVEF ≤ 40%) [2,6]. These measures capture different, although related, dimensions of HF severity and prognosis [2,6].

Oxidative stress has emerged as an important mechanism in HF pathophysiology. It results from an imbalance between reactive oxygen and nitrogen species and antioxidant defenses, leading to disrupted redox signaling and molecular injury [7]. Increased oxidative stress has been linked to endothelial dysfunction, inflammation, mitochondrial damage, myocardial remodeling, and progression of HF [8,9,10,11]. Among circulating biomarkers, malondialdehyde (MDA) reflects lipid peroxidation, while nitrates and nitrites (NOx) indicate nitric oxide-related redox activity. In contrast, superoxide dismutase (SOD), catalase (CAT), glutathione (GSH), and sulfhydryl (SH) groups represent key components of antioxidant defense [10,12,13,14,15]. Previous studies suggest that oxidative stress is increased in HF and may be related to greater disease severity and poorer outcomes [12,13,14].

Although NT-proBNP remains the most established biomarker in HF, it primarily reflects myocardial wall stress and neurohormonal activation rather than oxidative injury [2,3]. In contrast, the pattern of oxidative and antioxidant biomarkers across NYHA classes and LVEF-defined HF phenotypes is still not fully understood. It is particularly unclear whether individual markers are more strongly related to functional limitation, systolic dysfunction, or both. Therefore, this study examined circulating markers of oxidative and nitrosative stress in patients with HF classified according to NYHA functional class and LVEF, compared them with healthy controls, and assessed their correlations with NT-proBNP, their independent associations with HF severity, and their potential value as biomarkers of disease severity.

## 2. Materials and Methods

### 2.1. Study Design and Participants

This case–control study included 85 patients with heart failure (HF) and 33 healthy controls. Patients with HF were recruited between 26 January 2023 and 25 January 2025 at the Clinic for Internal Diseases, Military Hospital Novi Sad, Serbia. Eligible patients were screened during the recruitment period and enrolled if they fulfilled the predefined inclusion and exclusion criteria. Patients were not consecutively enrolled; enrollment was based on eligibility and availability during the recruitment period. The HF cohort included both hospitalized patients and outpatients with established chronic HF who were evaluated in a hospital-based internal medicine setting and were clinically stable at the time of enrollment. Patients were not included during an episode of acute decompensated HF, acute infection, or other acute inflammatory condition.

The screening process included review of medical records, direct physician–participant interview, clinical examination, and transthoracic echocardiographic assessment. The main inclusion criteria for the HF group were age ≥ 18 years, written informed consent, a diagnosis of chronic HF established by a cardiologist or internist, and absence of acute infection or inflammation at enrollment. The diagnosis of HF was based on the presence of typical symptoms and/or signs of HF, including exertional dyspnea, fatigue, reduced exercise tolerance, peripheral edema, pulmonary congestion, or other findings compatible with volume overload, together with objective evidence of structural and/or functional cardiac abnormality on transthoracic echocardiography. Echocardiographic assessment included evaluation of left ventricular systolic function, and LVEF was used to classify patients as having HF with preserved ejection fraction, mildly reduced ejection fraction, or reduced ejection fraction. NT-proBNP values were reviewed when available as supportive biochemical evidence of HF and were also analyzed as a study biomarker; however, inclusion in the HF group was based on the overall clinical diagnosis supported by echocardiographic findings rather than on NT-proBNP alone.

The main exclusion criteria for the HF group were chronic obstructive pulmonary disease and chronic renal failure because of their potential influence on the analyzed biological markers.

The control group was selected using a frequency-matching approach based on sex and body mass index (BMI). Control subjects were selected from military personnel undergoing periodic health examinations at the same institution. The main inclusion criteria for the control group were age ≥ 18 years, written informed consent, absence of significant health problems confirmed by clinical examination, no evidence of HF on echocardiography, and absence of acute infection or inflammation.

The control group was intended to serve as a clinically healthy reference group rather than as a fully matched cardiovascular comparator group. This source population was considered appropriate because control subjects were evaluated in the same institutional setting, underwent standardized clinical assessment, had no significant health problems confirmed by clinical examination, had no echocardiographic evidence of HF, and had no evidence of acute infection or inflammation at enrollment. Frequency matching was performed according to sex and BMI because these variables may influence oxidative stress biomarkers. However, age and comorbidity matching was not achieved. Therefore, comparisons between HF patients and controls were interpreted descriptively and cautiously, and not as evidence of HF-specific redox alterations independent of age, smoking status, cardiovascular risk factors, or comorbidity burden.

Sample size was calculated using G*Power v3.1.9.2. For analysis of variance, assuming an effect size of f = 0.33, α = 0.05, and power = 0.80, the required total sample size was 104 for two groups and 115 for five groups.

All participants received detailed information about the study and provided written informed consent before enrollment. The study was conducted in accordance with the Declaration of Helsinki and was approved by the Ethics Committee of the Military Hospital Novi Sad, Serbia (protocol code: 284-3; approval date: 25 January 2023).

### 2.2. Clinical Assessment and HF Severity Classification

Demographic characteristics, smoking status, medical history, comorbidities, disease duration, and treatment data were obtained from medical records and direct physician- participant interviews, with appropriate protection of personal data. Disease duration was defined as the time interval between the first documented diagnosis of HF in the medical record and the date of study enrollment. When the exact date of diagnosis was not clearly available in the medical record, disease duration was confirmed during the physician–participant interview and cross-checked with available clinical documentation whenever possible.

HF severity was assessed using both symptom-based and echocardiographic measures. Functional status was classified according to the New York Heart Association (NYHA) classification into classes I, II, III, and IV. Left ventricular systolic function was assessed by two-dimensional transthoracic echocardiography, and left ventricular ejection fraction (LVEF) was determined using Simpson’s biplane method in accordance with the recommendations of the American Society of Echocardiography [16]. Patients were categorized as having HF with preserved ejection fraction (HFpEF; LVEF ≥ 50%), HF with mildly reduced ejection fraction (HFmrEF; LVEF 41–49%), or HF with reduced ejection fraction (HFrEF; LVEF ≤ 40%).

For receiver operating characteristic (ROC) curve analysis, patients in the HF group were additionally categorized as NYHA class I/II versus III/IV and LVEF ≤ 40% versus > 40%.

### 2.3. Blood Sampling and Laboratory Analyses

Peripheral venous blood samples were collected in the morning after a 12 h overnight fast. Venipuncture was performed using a vacuum blood collection system (Becton Dickinson, Plymouth, UK) into tubes containing a clot activator for serum separation and heparinized tubes for plasma isolation. Samples were centrifuged at 2500 rpm for 15 min in a refrigerated centrifuge at 4 °C. Serum and plasma were separated into aliquots and stored at −80 °C until analysis. Immediately before analysis, aliquots were thawed at room temperature only once and gently mixed prior to measurement.

To reduce preanalytical variability, all samples were processed according to the same protocol. After centrifugation and before analysis, all samples were visually inspected for hemolysis, lipemia, turbidity, insufficient volume, improper labeling, and clotting in anticoagulated tubes. Samples with any of these findings, or with documented evidence of repeated freeze–thaw cycles, were excluded from the analysis. Sample aliquots intended for oxidative stress biomarker analyses were stored under identical conditions, and samples from patients with heart failure and controls were handled under the same preanalytical conditions. Direct exposure to light was minimized during the preparation and measurement of colorimetric and spectrophotometric assays, where applicable.

All analyses were performed at the Institute for Medical Research, Military Medical Academy, Belgrade, Serbia. Laboratory personnel were blinded to group assignment and HF severity categories during the assay procedures.

NT-proBNP was measured by electrochemiluminescence immunoassay on a cobas e 411 analyzer (Roche Diagnostics, Mannheim, Germany) and expressed as pg/mL. Calibration and quality-control procedures for NT-proBNP were performed according to the manufacturer’s instructions.

### 2.4. Determination of Oxidative Stress Biomarkers

The selected oxidative stress biomarkers were measured in samples using established spectrophotometric methods previously described in the literature, with additional practical assay conditions summarized below to improve reproducibility. Absorbance measurements for MDA and NOx were performed using an ELISA spectrophotometer (SynergyHT, BioTek, Winooski, VT, USA), whereas SOD and CAT activities were measured on an Ultrospec 2000 spectrophotometer (Pharmacia Biotech, Buckinghamshire, UK). SH groups and total GSH were analyzed using an ILAB 300 Plus biochemical analyzer (Instrumentation Laboratory, Milan, Italy).

Malondialdehyde (MDA), as a marker of lipid peroxidation, was determined in plasma using the thiobarbituric acid-reactive substances method (TBARS) and expressed as μmol/L [17]. In brief, MDA reacts with thiobarbituric acid to form a colored MDA–TBA adduct, the absorbance of which was measured at 492 nm. MDA concentrations were calculated using a standard calibration curve.

Total nitrates and nitrites (NOx), as indices of nitric oxide-related redox metabolism, were measured using the Griess reaction after reduction of nitrates to nitrites and expressed as μmol/L [18]. Briefly, nitrates were first reduced to nitrites, after which total nitrite concentration was determined by reaction with Griess reagents, producing a colored azo compound. Absorbance was measured at 492 nm, and NOx concentrations were calculated from a standard curve.

Total superoxide dismutase (SOD) activity was determined by the adrenaline autoxidation method and expressed as kU/L [19]. The assay is based on the ability of SOD to inhibit adrenaline autoxidation under alkaline conditions. The formation of adrenochrome was monitored spectrophotometrically at 480 nm for 3 min at 25 °C.

Catalase (CAT) activity was measured based on hydrogen peroxide decomposition and expressed as kU/L [20]. Samples were incubated with hydrogen peroxide in sodium–potassium phosphate buffer, and the reaction was stopped by the addition of ammonium molybdate, forming a colored complex measured at 405 nm.

Sulfhydryl (SH) groups were determined using the Ellman method and expressed as mmol/L [21]. In this assay, thiol groups react with 5,5′-dithiobis-(2-nitrobenzoic acid), DTNB, to form 5-thio-2-nitrobenzoic acid. Absorbance was measured at 412 nm, and SH group concentrations were calculated from a standard curve.

Total glutathione (GSH) concentration was measured by a DTNB–glutathione reductase recycling assay and expressed as μmol/L [22]. The rate of formation of 5-thio-2-nitrobenzoic acid was monitored spectrophotometrically at 412 nm and was proportional to total glutathione concentration.

Reagents used for the spectrophotometric oxidative stress assays were of analytical grade, and fresh working solutions were prepared according to the corresponding assay protocols. Calibration standards, reagent blanks, and internal control samples were included where appropriate for each assay.

All spectrophotometric oxidative stress assays were performed in duplicate. The mean value of accepted duplicate measurements was used for statistical analysis. Measurements were repeated when the relative difference between duplicate values exceeded the predefined acceptance limit of 10% or when technically implausible results were obtained. Analytical precision was monitored using reagent blanks, calibration standards, and internal control samples, where appropriate. Intra-assay coefficients of variation were 3.7% for MDA, 7.6% for NOx, 7.7% for SOD, 8.4% for CAT, 4.5% for SH groups, and 6.5% for GSH. Inter-assay coefficients of variation were 5.5% for MDA, 8.8% for NOx, 12.9% for SOD, 9.6% for CAT, 9.8% for SH groups, and 9.9% for GSH.

### 2.5. Statistical Analysis

Statistical analysis was performed using IBM SPSS Statistics for Windows (version 24.0; IBM Corp., Armonk, NY, USA). Data were presented as mean ± SD, median (range), or number (%), as appropriate. Differences between groups were assessed using Welch’s *t*-test, Pearson’s chi-square test, or Fisher’s exact test, as appropriate. Differences in oxidative stress biomarkers across NYHA classes and LVEF categories were analyzed using the Kruskal–Wallis test with Bonferroni-corrected post hoc comparisons. Correlations between NT-proBNP, HF severity indices, and oxidative stress biomarkers were evaluated using Spearman’s rank correlation coefficient. Ordinal logistic regression analysis was used to assess the associations of oxidative stress biomarkers and NT-proBNP with HF severity. ROC analysis was performed to evaluate their discriminatory ability for HF severity categories. A *p* value < 0.05 was considered statistically significant.

## 3. Results

### 3.1. Baseline Characteristics of the Study Population

Patients with HF differed markedly from controls in several baseline characteristics. They were older, more often smokers, and had higher systolic and diastolic blood pressure and heart rate, whereas sex distribution and BMI were similar between groups. In addition, the HF group had a substantially greater comorbidity burden and a higher prevalence of diabetes mellitus, arterial hypertension, rhythm disorders, and previous myocardial infarction (Table 1).

### 3.2. HF Severity Classification and Treatment

Within the HF group, the majority of patients were classified as NYHA class I/II, whereas 40.0% belonged to NYHA class III/IV. Based on LVEF, HFrEF was the predominant category, while HFpEF and HFmrEF were present in equal proportions. ACE inhibitors were used in nearly all patients, followed by diuretics and beta blockers, while cardiotonics were prescribed less frequently (Table 2).

### 3.3. Oxidative Stress Biomarkers According to NYHA Class and LVEF Category

**Figure 1:**
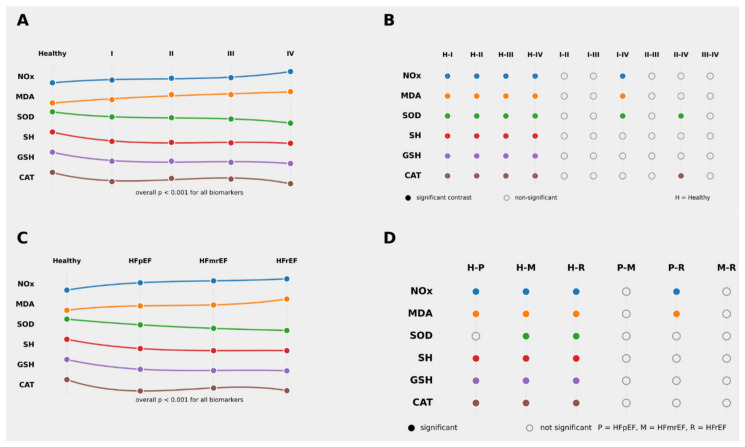
Summary of oxidative stress biomarker alterations according to NYHA class and LVEF category in heart failure. (**A**) Biomarker trends across NYHA classes relative to healthy controls. (**B**) Summary of pairwise post hoc comparisons between healthy controls and NYHA classes and among NYHA classes. (**C**) Biomarker trends according to LVEF category. (**D**) Summary of pairwise post hoc comparisons between healthy controls and LVEF categories and among LVEF categories. Filled circles indicate statistically significant differences, and open circles indicate non-significant differences. Overall group differences were assessed using the Kruskal–Wallis test, with Bonferroni-corrected post hoc comparisons. Abbreviations: CAT, catalase; GSH, glutathione; HF, heart failure; HFpEF, heart failure with preserved ejection fraction; HFmrEF, heart failure with mildly reduced ejection fraction; HFrEF, heart failure with reduced ejection fraction; LVEF, left ventricular ejection fraction; MDA, malondialdehyde; NOx, nitrates and nitrites; NYHA, New York Heart Association; SH, sulfhydryl groups; SOD, superoxide dismutase.

All oxidative stress biomarkers showed significant overall differences across NYHA classes and LVEF categories (all *p* < 0.001; Table 3A,B). These patterns are schematically summarized in Figure 1. Across NYHA classes, NOx and MDA increased with worsening HF severity, whereas SOD activity, SH groups, GSH, and CAT were lower in patients with HF than in healthy controls (Table 3A; Figure 1A). Post hoc analysis showed that NOx differed significantly between healthy controls and each NYHA class (all adjusted *p* < 0.001), while MDA was significantly higher in each NYHA class than in healthy controls (adjusted *p* = 0.034 for NYHA I and adjusted *p* < 0.001 for NYHA II–IV). Both NOx and MDA also differed between NYHA classes I and IV (adjusted *p* = 0.001 and adjusted *p* < 0.001, respectively). SOD activity was significantly lower in each NYHA class than in healthy controls (adjusted *p* = 0.025, 0.040, <0.001, and <0.001 for NYHA I–IV, respectively), with additional differences between NYHA classes I and IV and between II and IV (adjusted *p* = 0.022 and 0.006, respectively). SH groups and GSH showed a similar pattern, with significantly lower values in each NYHA class than in healthy controls (all adjusted *p* < 0.001). CAT values were also lower in HF, with significant differences between healthy controls and each NYHA class (adjusted *p* ≤ 0.006), as well as between NYHA classes II and IV (adjusted *p* = 0.002) (Table 3A; Figure 1B).

When classified according to LVEF, NOx and MDA were significantly higher in HFrEF than in HFpEF (adjusted *p* = 0.035 and 0.006, respectively), while the overall distribution of biomarker changes across LVEF categories is shown in Figure 1C and detailed in Table 3B. SH groups, GSH, and CAT differed significantly between healthy controls and all HF subtypes, but not among HF subtypes. For SOD, significant differences versus healthy controls were observed in the LVEF 41–49% and LVEF ≤ 40% groups, but not in the LVEF ≥ 50% group. Pairwise post hoc comparisons for the LVEF-based classification are summarized in Figure 1D. Overall, the figure illustrates a consistent pattern of increasing pro-oxidant markers (NOx and MDA) and decreasing antioxidant defense markers (SOD, SH groups, GSH, and CAT) with worsening functional status and reduced ejection fraction (Figure 1; Table 3B).

### 3.4. Correlation Analysis of NT-proBNP, HF Severity Indices, and Oxidative Stress Biomarkers

Spearman correlation analysis demonstrated that higher NT-proBNP levels were associated with greater HF severity, as reflected by a positive correlation with NYHA class and a negative correlation with LVEF. NYHA class showed positive correlations with NOx and MDA and negative correlations with CAT, GSH, and SOD, while LVEF was inversely associated with NOx and MDA and positively associated with SH groups and SOD. Among oxidative stress biomarkers, NOx was directly correlated with MDA and inversely correlated with antioxidant markers, whereas MDA showed negative correlations with CAT and SOD. Antioxidant biomarkers were positively intercorrelated, particularly SH groups, CAT, GSH, and SOD (Table 4).

### 3.5. Ordinal Logistic Regression Analysis of HF Severity and Oxidative Stress Biomarkers

Separate adjusted ordinal logistic regression analyses were performed to examine the associations of oxidative stress biomarkers and NT-proBNP with heart failure severity. NYHA class (I–IV) and LVEF category (from preserved to reduced left ventricular ejection fraction) were analyzed as dependent ordinal outcomes. Each biomarker and NT-proBNP was entered separately as a predictor, with adjustment for age, sex, BMI, disease duration, smoking, and diabetes mellitus (Table 5).

In these separate adjusted models, higher NOx, MDA, and NT-proBNP levels were associated with increased odds of worse NYHA class and more impaired LVEF category. Conversely, higher SH, CAT, GSH, and SOD levels were associated with lower odds of worse NYHA class and lower odds of more impaired LVEF category (Table 5).

Because all oxidative stress biomarkers were significant in the separate adjusted models, they were subsequently entered simultaneously into combined multivariable ordinal logistic regression models, again adjusted for age, sex, BMI, disease duration, smoking, and diabetes mellitus (Table 6). The variables included in the combined models were selected based on previous literature and the availability of relevant clinical data.

In the combined model with NYHA class as the dependent variable, only NOx remained independently associated with worse NYHA class. MDA, SH, CAT, GSH, and SOD were not independently associated with NYHA class.

In the combined model with LVEF category as the dependent variable, higher MDA levels were independently associated with greater odds of belonging to a more impaired LVEF category. SH, NOx, CAT, GSH, and SOD were not independently associated with LVEF category (Table 6).

### 3.6. ROC Curve Analysis of Oxidative Stress Biomarkers and NT-proBNP

Receiver operating characteristic (ROC) curve analysis was used to assess the ability of NT-proBNP, NOx, MDA, and SH levels to discriminate between NYHA class I/II and III/IV, as well as between LVEF ≤ 40% and >40%. Optimal cutoff values were determined using the Youden index.

For discrimination between NYHA class I/II and III/IV, NT-proBNP showed the best performance, followed by NOx and MDA, whereas SH groups were not discriminatory. For discrimination between LVEF ≤ 40% and >40%, MDA showed the highest discriminatory ability, followed closely by NT-proBNP and NOx, while SH groups again showed no meaningful discriminatory performance (Table 7).

Pairwise ROC curve comparisons for discrimination between NYHA class I/II and III/IV showed that NT-proBNP had a larger AUC than NOx, MDA, and SH, while NOx also outperformed SH. No significant differences were observed between NOx and MDA or between MDA and SH (Table 8).

For discrimination between LVEF ≤ 40% and >40%, MDA, NT-proBNP, and NOx showed comparable discriminatory performance, whereas SH groups had limited value. Pairwise ROC curve comparisons confirmed the absence of significant differences among MDA, NT-proBNP, and NOx, while each of these markers outperformed SH groups (Table 8).

## 4. Discussion

In this case–control study, circulating markers of oxidative and nitrosative stress were associated with both symptomatic and systolic severity of HF. Compared with healthy controls, patients with HF had higher NOx and MDA concentrations and lower SOD activity, SH groups, GSH, and CAT. After adjustment for clinical covariates, all examined oxidative stress biomarkers were associated with both NYHA class and LVEF category. However, when these biomarkers were entered simultaneously into combined multivariable models, the pattern became more selective: NOx remained independently associated with NYHA class, whereas MDA remained independently associated with LVEF category, while SH showed only a borderline inverse association with LVEF. ROC analysis broadly supported this pattern, with NOx showing the best discriminatory performance among oxidative stress biomarkers for symptomatic severity and MDA performing best for reduced LVEF. Nevertheless, NT-proBNP remained the strongest overall discriminator, particularly for NYHA class, confirming its central role in HF assessment [2,3,6].

The case–control findings should be interpreted in light of baseline differences between the study groups. Patients with HF were older and had a substantially greater burden of comorbidities than healthy controls. Because aging and cardiometabolic disease can influence oxidative stress and inflammation [23,24,25,26], while vascular dysfunction is closely linked to altered nitric oxide signaling in cardiovascular disease and HF [27,28,29,30,31,32], the observed biomarker differences cannot be attributed exclusively to HF itself on the basis of the between-group comparison alone. Accordingly, although the observed biomarker profile is compatible with HF-related redox imbalance, the case–control analysis cannot fully disentangle effects attributable to HF from those related to age, systemic inflammation, and comorbidity burden. In this context, the adjusted regression analyses are particularly informative, as they provide a more conservative estimate of the independent association between oxidative stress biomarkers and HF severity.

Overall, these findings support the presence of increased oxidative and nitrosative stress together with reduced antioxidant defense in HF. This interpretation is consistent with current understanding of metabolic remodeling in the failing myocardium, including impaired substrate utilization, reduced metabolic flexibility, mitochondrial dysfunction, and disturbed ROS/RNS signaling, all of which can promote progressive redox imbalance and myocardial injury [33]. In this context, the observed increase in NOx and MDA, together with lower antioxidant parameters, is in line with previous reports of increased systemic oxidative burden in HF [7,8,11,12].

The present findings should also be interpreted within the broader context of chronic low-grade inflammation in HF. Oxidative/nitrosative stress and inflammatory activation are closely interconnected processes that may jointly contribute to endothelial dysfunction, mitochondrial impairment, myocardial remodeling, and HF progression. As highlighted in the recent review [34], inflammation remains a mechanistically important but incompletely resolved component of chronic HF pathophysiology. Therefore, the associations of NOx with symptomatic severity and MDA with systolic dysfunction may reflect not only isolated redox imbalance, but also the wider inflammatory, endothelial, and cardiometabolic milieu of HF.

One of the main findings was that NOx and MDA were the oxidative stress biomarkers most consistently related to HF severity, although their associations differed depending on the analytical context. After adjustment for clinical covariates, both biomarkers were associated with worse NYHA class and more impaired LVEF category, together with the antioxidant markers. In the combined models, however, NOx remained independently associated only with NYHA class, suggesting a closer relationship with symptomatic and functional impairment, whereas MDA remained independently associated with LVEF category, suggesting a stronger link with systolic dysfunction. This distinction is clinically relevant because NYHA class and LVEF reflect related, but not identical, dimensions of HF severity [2,3,6]. Overall, these findings suggest that NOx may better capture the functional and systemic burden of HF, whereas MDA may more closely reflect oxidative damage linked to ventricular systolic impairment [13,35,36,37]. ROC analysis supported this pattern: among the oxidative stress biomarkers, NOx performed best for discrimination between NYHA I/II and III/IV, whereas MDA showed the highest AUC for LVEF ≤ 40% versus > 40%.

The findings related to MDA are consistent with previous studies indicating that lipid peroxidation is an important component of oxidative stress in HF. Polidori et al. reported higher MDA concentrations together with lower antioxidant levels in patients with more advanced NYHA class [13]. Radovanović et al. showed that markers of oxidative damage, including MDA, were associated with worse chronic HF and adverse prognosis [35]. Romuk et al. likewise identified MDA as an independent predictor of unfavorable outcomes in chronic HF [36]. More recent evidence in elderly patients with recurrent HF after coronary stenting also linked higher MDA levels with higher NYHA class and lower LVEF [37]. Taken together with our findings, these data support MDA as a marker of oxidative injury related both to clinical severity and to ventricular dysfunction.

The interpretation of NOx is less straightforward because both increased and decreased nitric oxide metabolite levels have been reported in cardiovascular disease and HF. Nitric oxide has complex and sometimes opposing roles in HF pathophysiology. Reduced NO bioavailability contributes to endothelial dysfunction, impaired vasodilation, and abnormal ventricular–vascular interactions, whereas excessive NO production, particularly via inducible nitric oxide synthase, may promote nitrosative stress, mitochondrial injury, cardiomyocyte dysfunction, and adverse remodeling [27,28,29,30,31,32,38,39,40]. In our study, NOx concentrations were higher in HF patients, increased with worsening NYHA class, and remained independently associated with symptomatic severity, which is compatible with enhanced nitrosative stress in more advanced HF. Still, this finding should be interpreted cautiously. Chirinos et al. reported lower circulating nitric oxide metabolites in HFpEF than in HFrEF and emphasized that circulating NOx reflects not only endogenous NO biology but also clearance and dietary nitrate/nitrite intake [41]. In addition, nitrate-related therapeutic pathways may influence circulating NO metabolites [42]. Therefore, circulating NOx may be better interpreted as an indirect systemic marker of nitric oxide-related redox imbalance than as a myocardium-specific indicator.

All antioxidant parameters were lower in HF than in controls, further supporting the presence of reduced antioxidant reserve in chronic HF. After adjustment for clinical covariates, SH, CAT, GSH, and SOD were associated with both NYHA class and LVEF category, indicating that antioxidant depletion parallels both symptomatic and systolic severity. However, these associations were attenuated when the antioxidant markers were evaluated together with the other oxidative stress parameters. Under those conditions, none remained independently associated with NYHA class. This pattern suggests that antioxidant depletion may be a general feature of HF-related redox imbalance, whereas the prognostic information provided by individual antioxidant markers may substantially overlap with that carried by other components of the oxidative stress profile. The overall direction of our findings remains consistent with previous reports describing altered SOD activity, catalase-related changes, and thiol depletion in HF [14,43,44,45,46,47].

The correlation analysis further supports the internal consistency of these findings. NT-proBNP showed the expected positive association with HF severity, reinforcing its role as a reference biomarker [2,3,6]. Among the oxidative stress biomarkers, NOx demonstrated the most consistent relationship with severity indices and inverse correlations with antioxidant parameters, whereas MDA showed a similar, although somewhat less uniform, pattern. At the same time, the substantial intercorrelations among NOx, MDA, GSH, SOD, CAT, and SH groups indicate that these biomarkers reflect not isolated pathways, but overlapping components of a broader redox network. This overlap likely explains why several biomarkers were significant after covariate adjustment when analyzed individually, but lost significance when evaluated simultaneously in the combined multivariable models. Clinically, these observations do not suggest that oxidative stress biomarkers can replace natriuretic peptides in HF assessment, because NT-proBNP remains central to contemporary HF diagnostic and risk-assessment algorithms [2,3,6]. Rather, NOx and MDA may provide complementary mechanistic information reflecting different aspects of redox imbalance in relation to HF severity [7,8,11,12,33,48]. This interpretation is supported by our ROC analysis, in which NT-proBNP remained the strongest overall discriminator, particularly for NYHA class, whereas NOx and MDA showed only moderate discriminatory performance (Table 7). Therefore, the potential adjunctive role of these biomarkers should be considered exploratory, and it requires confirmation in larger prospective studies.

Several limitations should be acknowledged. First, a major limitation of the case–control comparison is the substantial imbalance between the HF and control groups. Although controls were frequency-matched for sex and BMI, they were considerably younger and had no documented comorbidities, whereas patients with HF had a high burden of smoking, hypertension, diabetes mellitus, rhythm disorders, previous myocardial infarction, and other comorbid conditions. The use of young, comorbidity-free military personnel as controls may have introduced selection bias and limits causal attribution in the between-group comparisons. Since age, smoking, cardiometabolic risk factors, inflammation, and comorbidities may independently affect oxidative stress biomarkers, the observed differences between HF patients and controls cannot be attributed exclusively to HF. Although regression models were adjusted for relevant clinical covariates, including age, sex, BMI, smoking, diabetes, and disease duration where applicable, statistical adjustment cannot fully compensate for the substantial baseline differences between HF patients and controls. Therefore, the control group should be interpreted as a clinically healthy reference group rather than as a fully matched cardiovascular comparator group, and between-group comparisons should be considered descriptive and exploratory rather than evidence of HF-specific redox alterations [23,24,25,26]. Future studies should include age- and cardiometabolic-risk-matched control groups to reduce selection bias and improve causal interpretation.

Second, the modest size of some HF severity subgroups should also be acknowledged. Although the total sample size was consistent with the planned power calculation, the number of patients in NYHA class IV and in the HFpEF and HFmrEF categories was relatively small. This may reduce the precision of subgroup-level estimates and limit the robustness of post hoc comparisons across NYHA classes and LVEF categories. Therefore, subgroup-level findings, adjusted regression models, and ROC analyses should be interpreted as exploratory and hypothesis-generating, and they require confirmation in larger cohorts with more balanced representation across HF severity categories.

Third, a further important limitation concerns the interpretation of circulating NOx concentrations. Dietary nitrate and nitrite intake was not standardized or recorded, although it may substantially influence circulating nitrate/nitrite levels. In addition, NOx concentrations may be affected by renal clearance, nitrate-related therapies, and other cardiovascular medications that influence endothelial function or nitric oxide-related pathways, including ACE inhibitors and other vasomodulatory treatments. Therefore, NOx should be interpreted as an indirect systemic marker of nitric oxide-related redox imbalance rather than as a myocardium-specific indicator. Future studies should include dietary nitrate control or recording, more detailed medication adjustment, and standardized preanalytical conditions for NOx measurement [41,42].

## 5. Conclusions

This study showed that HF is accompanied by increased oxidative and nitrosative stress together with reduced antioxidant defense, and that these alterations are associated with both NYHA class and LVEF. Among the oxidative biomarkers examined, NOx was most consistently related to functional severity, whereas MDA showed an independent association with LVEF category in adjusted analyses. Although NT-proBNP remained the strongest overall discriminator, NOx and MDA may provide complementary mechanistic information on redox-related pathophysiology across HF severity categories. These findings should be considered exploratory, and require confirmation in larger prospective studies with better-matched control groups and more detailed control of dietary, therapeutic, inflammatory, and cardiometabolic confounders.

## Figures and Tables

**Table 1:** Baseline demographic and clinical characteristics of the study population.

Variable	Control	HF	*p* Value *
	*n* = 33	*n* = 85	
Sex, n (%)			0.406
Male	24 (72.7%)	55 (64.7%)	
Female	9 (27.3%)	30 (35.3%)	
Age, years, mean ± SD	36.0 ± 11.1	63.1 ± 9.7	<0.001
BMI, kg/m^2^, mean ± SD	25.4 ± 3.7	25.1 ± 2.8	0.675
Smoking, n (%)			0.021
Smoker	12 (36.4%)	51 (60.0%)	
Non-smoker	21 (63.6%)	34 (40.0%)	
Blood pressure, mm Hg, mean ± SD			
Systolic	125.3 ± 6.9	163.6 ± 17.9	<0.001
Diastolic	81.1 ± 9.7	99.2 ± 11.2	<0.001
Heart rate, beats/min, mean ± SD	94 ± 13	100 ± 12	0.025
Number of comorbidities, n (%)			<0.001
None	33 (100.0%)	0 (0.0%)	
One	0 (0.0%)	20 (23.5%)	
Two	0 (0.0%)	24 (28.2%)	
Three	0 (0.0%)	16 (18.8%)	
Four or more	0 (0.0%)	25 (29.4%)	
Type of comorbidity, n (%)			
Diabetes mellitus	0 (0.0%)	51 (60.0%)	<0.001
Arterial hypertension	0 (0.0%)	66 (77.6%)	<0.001
Rhythm disorder	0 (0.0%)	22 (25.9%)	<0.001
Myocardial infarction	0 (0.0%)	53 (62.4%)	<0.001

* Continuous variables were compared using *t*-test. Categorical variables were compared using Pearson’s chi-square test or Fisher’s exact test, as appropriate. Abbreviations: BMI, body mass index; HF, heart failure; SD, standard deviation.

**Table 2:** Classification and treatment of patients with HF.

Variable	n = 85
NYHA class, n (%)	
I	23 (27.1%)
II	28 (32.9%)
III	19 (22.4%)
IV	15 (17.6%)
LVEF classification, n (%)	
≥50%	20 (23.5%)
41–49%	20 (23.5%)
≤40%	45 (52.9%)
Treatment, n (%)	
ACE inhibitors	84 (98.8%)
Beta blockers	46 (54.1%)
Diuretics	47 (55.3%)
Cardiotonics	22 (25.9%)

Abbreviations: ACE, angiotensin-converting enzyme; HF, heart failure; LVEF, left ventricular ejection fraction; NYHA, New York Heart Association.

**Table 3:** (**A**). Oxidative stress biomarkers according to NYHA class. (**B**). Oxidative stress biomarkers according to LVEF category.

**(A)**						
**Variable**	**Healthy** **(n = 33)**	**NYHA I** **(n = 23)**	**NYHA II** **(n = 28)**	**NYHA III** **(n = 19)**	**NYHA IV** **(n = 15)**	***p*** **-Value**
NOx (μmol/L)	9.24 (5.71–13.35)	25.71 (15.11–82.33)	37.05 (15.67–72.89)	40.41 (19.00–86.22)	77.33 (22.89–99.94)	<0.001
MDA (μmol/L)	94.29 (65.71–111.43)	135.43 (84.00–214.29)	198.29 (94.29–248.57)	198.29 (100.00–294.29)	235.71 (146.86–288.57)	<0.001
SOD (kU/L)	852.18 (626.09–1078.26)	591.30 (243.48–1013.33)	552.55 (68.57–1226.67)	480.00 (137.14–1733.33)	274.29 (102.86–986.67)	<0.001
SH (mmol/L)	0.44 (0.30–0.65)	0.25 (0.16–0.44)	0.23 (0.18–0.61)	0.22 (0.16–0.62)	0.21 (0.18–0.31)	<0.001
GSH (μmol/L)	22.94 (15.05–31.50)	11.47 (7.17–24.60)	11.47 (7.17–29.39)	10.04 (5.81–31.54)	8.60 (7.17–15.05)	<0.001
CAT (kU/L)	6.63 (5.15–9.82)	3.20 (1.97–8.60)	4.42 (1.72–11.80)	4.17 (1.96–7.13)	2.46 (1.47–3.44)	<0.001
**(B)**						
**Variable**	**Healthy** **(n = 33)**	**LVEF ≥ 50%** **(n = 20)**	**LVEF 41–49%** **(n = 20)**	**LVEF ≤ 40%** **(n = 45)**	***p*** **-Value**	
NOx (μmol/L)	9.24 (5.71–13.35)	30.41 (15.11–82.33)	35.98 (19.00–94.00)	41.78 (17.33–99.94)	<0.001	
MDA (μmol/L)	94.29 (65.71–111.43)	141.14 (84.00–226.86)	151.43 (95.30–248.57)	214.29 (89.71–294.29)	<0.001	
SOD (kU/L)	852.18 (626.09–1078.26)	640.00 (243.48–1226.67)	506.67 (68.57–1066.67)	428.57 (102.86–1733.33)	<0.001	
SH (mmol/L)	0.44 (0.30–0.65)	0.26 (0.16–0.58)	0.22 (0.18–0.61)	0.22 (0.16–0.62)	<0.001	
GSH (μmol/L)	22.94 (15.05–31.50)	12.19 (7.17–29.39)	10.75 (7.17–25.81)	10.47 (5.81–31.54)	<0.001	
CAT (kU/L)	6.63 (5.15–9.82)	2.95 (1.97–8.12)	3.93 (1.47–7.48)	3.07 (1.47–11.80)	<0.001	

Values are presented as median (range). Overall group comparisons were performed using the Kruskal–Wallis test. Abbreviations: CAT, catalase; GSH, glutathione; LVEF, left ventricular ejection fraction; MDA, malondialdehyde; NOx, nitrates and nitrites; SH, sulfhydryl groups; SOD, superoxide dismutase; NYHA, New York Heart Association.

**Table 4:** Spearman correlations between NT-proBNP, HF severity indices, and oxidative stress biomarkers.

Variable	NT-proBNP	NYHA	LVEF	NOx	MDA	SH	CAT	GSH
NYHA	0.93 (<0.001)							
LVEF	−0.56 (<0.001)	−0.60 (<0.001)						
NOx	0.61 (<0.001)	0.61 (<0.001)	−0.51 (<0.001)					
MDA	0.57 (<0.001)	0.53 (<0.001)	−0.45 (<0.001)	0.61 (<0.001)				
SH	−0.11 (0.331)	−0.16 (0.140)	0.25 (0.025)	−0.48 (<0.001)	−0.14 (0.213)			
CAT	−0.18 (0.104)	−0.24 (0.030)	0.16 (0.156)	−0.29 (0.008)	−0.26 (0.016)	0.45 (<0.001)		
GSH	−0.23 (0.036)	−0.25 (0.022)	0.20 (0.072)	−0.39 (<0.001)	−0.13 (0.227)	0.46 (<0.001)	0.55 (<0.001)	
SOD	−0.38 (<0.001)	−0.42 (<0.001)	0.40 (<0.001)	−0.56 (<0.001)	−0.37 (<0.001)	0.56 (<0.001)	0.48 (<0.001)	0.65 (<0.001)

Data are presented as Spearman’s rho (*p* value). Abbreviations: CAT, catalase; GSH, glutathione; LVEF, left ventricular ejection fraction; MDA, malondialdehyde; NOx, nitrates and nitrites; NT-proBNP, N-terminal pro-B-type natriuretic peptide; NYHA, New York Heart Association; SH, sulfhydryl groups; SOD, superoxide dismutase.

**Table 5:** Separate adjusted ordinal logistic regression analyses according to NYHA and LVEF classification.

Dependent Variable	NYHA Class * OR (95% CI)	LVEF Category * OR (95% CI)
NOx (μmol/L)	1.11 (1.08–1.14) *p* < 0.001	1.07 (1.04–1.11) *p* < 0.001
MDA (μmol/L)	1.02 (1.02–1.03) *p* < 0.001	1.02 (1.01–1.03) *p* < 0.001
SH (per 0.1 mmol/L)	0.51 (0.36–0.73) *p* < 0.001	0.41 (0.28–0.60) *p* < 0.001
CAT (kU/L)	0.59 (0.48–0.73) *p* < 0.001	0.67 (0.54–0.83) *p* < 0.001
GSH (μmol/L)	0.88 (0.82–0.93) *p* < 0.001	0.88 (0.82–0.94) *p* < 0.001
SOD (per 10 kU/L)	0.97 (0.96–0.99) *p* < 0.001	0.98 (0.97–0.99) *p* = 0.003
NT-proBNP (per 100 pg/mL)	1.22 (1.11–1.35) *p* < 0.001	1.02 (1.00–1.03) *p* = 0.012

* Adjusted for age, sex, BMI, smoking, diabetes, and disease duration. SH was expressed per 0.1 mmol/L increase, SOD per 10 kU/L increase, and NT-proBNP per 100 pg/mL increase for interpretability. NOx: nitrates and nitrites; MDA: malondialdehyde; SOD: superoxide dismutase; SH: sulfhydryl groups; GSH: glutathione; CAT: catalase; NYHA: New York Heart Association functional class; LVEF: left ventricular ejection fraction category.

**Table 6:** Combined multivariable ordinal logistic regression models according to NYHA class and LVEF category.

	NYHA Class * OR (95% CI)	LVEF Category * OR (95% CI)
NOx (μmol/L)	1.07 (1.04–1.11) *p* < 0.001	1.02 (0.99–1.06) *p* = 0.179
MDA (μmol/L)	0.99 (0.98–1.00) *p* = 0.07	1.02 (1.00–1.03) *p* = 0.022
SH (per 0.1 mmol/L)	1.15 (0.54–2.45) *p* = 0.712	0.43 (0.18–1.03) *p* = 0.059
CAT (kU/L)	1.18 (0.83–1.67) *p* = 0.361	1.36 (0.89–2.09) *p* = 0.159
GSH (μmol/L)	1.02 (0.88–1.19) *p* = 0.806	(0.86–1.19) *p* = 0.893
SOD (per 10 kU/L)	(0.99–1.03) *p* = 0.257	(0.98–1.03) *p* = 0.685

* Adjusted for age, sex, BMI, smoking, diabetes, and disease duration. NOx: nitrates and nitrites; MDA: malondialdehyde; SOD: superoxide dismutase; SH: sulfhydryl groups; GSH: glutathione; CAT: catalase; NYHA: New York Heart Association functional class; LVEF: left ventricular ejection fraction category. SH was expressed per 0.1 mmol/L increase and SOD per 10 kU/L increase for interpretability.

**Table 7:** ROC curve analysis of NT-proBNP and oxidative stress biomarkers for discrimination of HF severity categories.

**(A). NYHA Class I/II Versus III/IV**						
**Variable**	**AUC**	**95% CI**	***p* Value**	Optimal Cutoff *	**Sensitivity, %**	**Specificity, %**
NT-proBNP	0.966	0.903–0.993	<0.001	>2522	88.2	98.0
NOx	0.782	0.679–0.864	<0.001	>42.18	61.8	88.2
MDA	0.723	0.615–0.815	<0.001	>221.4	47.1	92.2
SH	0.611	0.499–0.715	0.075	≤0.22	64.7	60.8
**(B). LVEF ≤ 40% Versus >40%**						
**Variable**	**AUC**	**95% CI**	***p* Value**	Optimal Cutoff *	**Sensitivity, %**	**Specificity, %**
MDA	0.751	0.644–0.839	<0.001	>180	71.1	69.2
NT-proBNP	0.746	0.639–0.835	<0.001	>3095.4	54.5	89.7
NOx	0.726	0.617–0.817	<0.001	>41.22	53.3	87.2
SH	0.573	0.460–0.680	0.253	≤0.22	57.8	61.5

* Optimal cutoff values were determined using the Youden index. Abbreviations: AUC, area under the curve; CI, confidence interval; HF, heart failure; LVEF, left ventricular ejection fraction; MDA, malondialdehyde; NOx, nitrates and nitrites; NT-proBNP, N-terminal pro-B-type natriuretic peptide; ROC, receiver operating characteristic; SH, sulfhydryl groups.

**Table 8:** Pairwise comparison of ROC curves for discrimination of HF severity categories.

**(A). NYHA Class I/II Versus III/IV**				
**Comparison**	**Difference Between AUCs**	**95% CI**	**z Statistic**	***p* Value**
NT-proBNP vs. NOx	0.185	0.0816 to 0.287	3.515	0.0004
NT-proBNP vs. MDA	0.243	0.143 to 0.344	4.744	<0.0001
NT-proBNP vs. SH	0.355	0.227 to 0.483	5.448	<0.0001
NOx vs. MDA	0.0588	−0.0487 to 0.166	1.072	0.2835
NOx vs. SH	0.171	0.0608 to 0.281	3.043	0.0023
MDA vs. SH	0.112	−0.0383 to 0.262	1.460	0.1443
**(B). LVEF ≤ 40% Versus >40%**				
**Comparison**	**Difference Between AUCs**	**95% CI**	**z Statistic**	***p* Value**
NT-proBNP vs. NOx	0.0202	−0.0880 to 0.128	0.366	0.7142
NT-proBNP vs. MDA	0.00484	−0.108 to 0.118	0.0842	0.9329
NT-proBNP vs. SH	0.173	0.0133 to 0.333	2.123	0.0338
NOx vs. MDA	0.0251	−0.0789 to 0.129	0.473	0.6364
NOx vs. SH	0.153	0.0334 to 0.272	2.509	0.0121
MDA vs. SH	0.178	0.0233 to 0.332	2.256	0.0241

Abbreviations: AUC, area under the curve; CI, confidence interval; HF, heart failure; LVEF, left ventricular ejection fraction; MDA, malondialdehyde; NOx, nitrates and nitrites; NT-proBNP, N-terminal pro-B-type natriuretic peptide; ROC, receiver operating characteristic; SH, sulfhydryl groups. Note: Pairwise ROC curve comparisons were performed using the DeLong method.

## Data Availability

The data presented in this study are available on request from the corresponding author due to hospital policies.
